# Melanocortin agonists stimulate lipolysis in human adipose tissue explants but not in adipocytes

**DOI:** 10.1186/s13104-015-1539-4

**Published:** 2015-10-12

**Authors:** Cathrine Laustrup Møller, Steen B. Pedersen, Bjørn Richelsen, Kilian W. Conde-Frieboes, Kirsten Raun, Kevin L. Grove, Birgitte Schjellerup Wulff

**Affiliations:** Diabetes and Obesity Biology, Novo Nordisk A/S, 2760 Maaloev, Denmark; Protein and Peptide Chemistry 3, Novo Nordisk A/S, 2760 Maaloev, Denmark; Type 2 Diabetes, Novo Nordisk A/S, 2760 Maaloev, Denmark; Department of Endocrinology MEA, Aarhus University Hospital, 8000 Aarhus, Denmark; Diabetes, Obesity and Metabolism, Oregon National Primate Research Centre, Oregon Health & Science University, Portland, OR 97006 USA; Steno Diabetes Center, Niels Steensensvej 2-4, 2820 Gentofte, Denmark; Obesity Research, Novo Nordisk A/S, Seattle, WA 98109 USA

**Keywords:** Melanocortin receptor, MC5R, MC4R, Lipolysis, NEFA, Glycerol, Propranolol, White adipose tissue

## Abstract

**Background:**

The central melanocortin system is broadly involved in the regulation of mammalian nutrient utilization. However, the function of melanocortin receptors (MCRs) expressed directly in peripheral metabolic tissues is still unclear. The objective of this study was to investigate the lipolytic capacity of MC1-5R in differentiated adipocytes versus intact white adipose tissue.

**Results:**

Non-selective MCR agonist α-MSH, MC5R-selective agonist PG-901 and MC4R-selective agonist LY2112688 significantly stimulated lipolysis in intact white adipose tissue, whereas stimulation of MCRs in differentiated adipocytes failed to do so. The lipolytic response of MC5R was decreased in intact human white adipose tissue when co-treating with β-adrenergic antagonist propranolol, suggesting that the effect may be dependent on neuronal innervation via noradrenalin release.

**Conclusion:**

When developing an anti-obesity therapeutic drug with selective MC4R/MC5R properties, effects on lipolysis in white adipose tissue may be physiologically relevant.

## Background

The melanocortin receptors (MCRs) are G-protein coupled receptors (family A), which are stimulated by endogenous proopiomelanocortin (POMC) derived agonists including ACTH and α-, β- and γ-MSH. The receptor system also contains melanocortin receptor-associated protein (MRAP) and MRAP2, as well as two antagonists, agouti-related peptide (AgRP) and agouti/agouti signaling protein (ASIP).

The melanocortin system has an essential function in the regulation of satiety, glucose homeostasis and energy expenditure [1, 2]. These effects are mainly mediated by MC4R expressed in the hypothalamus and the brainstem [3]. MC4R knockout mice have an increased lean body mass and fat mass, hyperphagia and disturbances in the metabolic response to overnutrition [3, 4]. Likewise, humans with MC4R mutations develop obesity [5], and in severe, early onset childhood obesity, the frequency of mutations in the MC4R locus causing decreased functionality is 4–6 % [6]. It has been shown that MC4R mRNA is expressed in autonomic control regions projecting to postganglionic neurons innervating peripheral metabolic tissues [3, 7–10]. Through sympathetic nerve innervation, CNS-MC4R has been shown to increase lipolysis in WAT [11, 12], insulin sensitivity in muscle [12, 13], thermogenesis in brown adipose tissue [10], fatty acid oxidation in liver [14] and decrease insulin release in pancreatic β-cells [15].

It is still rather unclear as to what extent peripherally expressed MCRs regulate metabolism. Notably, melanocortin peptides such as ACTH, β-lipotrophin, β-endorphin and α-MSH are released from the pituitary gland to the blood stream [16–19], raising the possibility that there may be peripheral actions of melanocortins on metabolic systems. In addition, POMC expression has been identified in peripheral tissues such as the skin, testes, ovaries, placenta, adrenal glands, gastrointestinal tract, immune cells and tumors [20, 21]. The peripheral melanocortin system was initially examined for its effects on pigmentation and adrenal function mediated by MC1R and MC2R. MC1R action is primarily linked to melanocytes where α-MSH stimulation induces synthesis of the brown/black pigment eumelanin [22]. MC2R is expressed within the adrenal cortex where it regulates glucocorticoid production when activated by ACTH [23] and plays a pivotal role in the regulation of the hypothalamic–pituitary–adrenal axis. More recently, α-MSH has been shown to inhibit proliferation and adipogenesis in human preadipocytes mediated by MC1R, which is antagonized by agouti/ASIP [24]. Agouti/ASIP is a high affinity antagonist on MC1R and increased ectopic expression of this protein is associated phenotypically with yellow fur (mice), late onset obesity, hyperphagia, increased growth and non-insulin dependent diabetes [24]. Notably, ASIP mRNA is increased in subcutaneous WAT from type II diabetic patients and is suggested to be a paracrine hormone that may induce proliferation and adipogenesis [25]. MC1R mRNA expression is shown to be co-localized with macrophages in adipose tissue of obese subjects, suggesting an inflammatory function of this receptor [26]. Furthermore, α-MSH has been shown to decrease the expression and release of leptin from adipocytes as a part of a negative feedback loop to the CNS, which is antagonized by AgRP [27]. In rats, this effect is likely mediated by MC4R and MC5R [28]. Furthermore, a novel study by Panaro et al. shows that MC4R is expressed in cells of the gastrointestinal system and that MC4R agonist administration elevates plasma PYY and GLP-1 in mice [29].

MC5R is extensively distributed in the periphery [30, 31], and is reported to regulate sebaceous gland secretion [32] and fatty acid oxidation in skeletal muscle [33]. The melanocortin system has a well-established lipolytic effect in rodent adipose tissues [34–37]. Studies in murine primary adipocytes and differentiated 3T3-L1 adipocytes show a direct lipolytic effect of MC2R and MC5R when stimulated by various endogenous and synthetic melanocortin agonists [35, 37, 38]. Moreover, an early study by Richter et al. showed a direct effect of α-MSH and ACTH on lipolysis in rabbit adipocytes [39]. However, it is unclear whether melanocortins have these actions in human adipose tissue. Expression of MC1R, MC4R and MC5R has been suggested in the adipose tissue of human obese subjects and these receptors may reflect an aspect of the pathophysiology of human obesity [26, 40, 41].

This study was undertaken to determine the lipolytic capacity of melanocortin receptors in differentiated human adipocytes versus intact adipose tissue. Initially, binding studies of selective- and non-selective MCR compounds were conducted on MC1-5R. We examined the possibility that MCR-mediated lipolysis is dependent on neuronal innervation via noradrenalin release in human adipose tissue.

## Methods and procedures

### Test compounds

α-MSH (Bachem), ACTH (Bachem), PG-901, LY2112688 (prepared in-house using standard peptide synthesis protocol); isoproterenol and propranolol were purchased from Sigma (Sigma-Aldrich Co. LLC).

### Materials

Qiagen RNeasy mini kit was used for extraction of RNA from adipocytes. cDNA was synthesized using iScript™ (Biorad). Gene expression assays (ABI Life Technology) were used in PCR. PrimePCR DNA contamination assay from Biorad was used to determine genomic contamination of cDNA. Human subcutaneous adipocytes were purchased from Zenbio Inc. 199/EBSS Hyclone medium (Thermo Scientific) was used in incubation of human adipose tissue explants. NEFA-HR (2) kit from Wako and Glycerol colorimetric assay kit from CaymanChemicals was employed when measuring lipolysis.

### Preparation of human WAT explants

Adipose tissue biopsies used for stimulation of lipolysis were taken from the subcutaneous abdominal region by needle aspiration as previously described [42]. The experimental group consisted of 6 non-diabetic females with a normal/preobese BMI (average BMI = 25.5). All biopsies were transported in sterile containers to the laboratory within 30 min after removal. The biopsies were washed repeatedly with isotonic saline and were used for subsequent culture.

### Ethical consideration

Experiments with stimulation of human white adipose tissue explants at Aarhus University Hospital were approved by the local Ethics Committee, and the subjects provided written informed consent.

### RNA extraction and cDNA synthesis

Qiagen RNeasy mini kit was used for extraction of RNA from adipocytes purchased at ZenBio. DNAse treatment was used in RNA extraction from adipocytes. The RNA was quantified measuring absorbance at 260 nm by Nanodrop and the purity of the RNA was indicated by a 260/280 ratio of 1.8 or higher to ensure no protein contamination. Finally, the integrity of the RNA was checked by visual inspection of the two ribosomal RNAs 18S and 28S on an agarose gel. iScript™ cDNA synthesis reactions were incubated 5 min at 25 °C, 30 min at 42 °C and 5 min at 85 °C. We found no genomic contamination of cDNA (using Biorad DNA contamination assay).

### PCR

In standard PCR, RNA was extracted from adipocytes isolated from 3 obese males (purchased from Zenbio) and pooled (cDNA 1:10 from 1 μg RNA, 45 cycles). Each reaction was prepared using 13 μl of Platinum PCR supermix (Invitrogen), 5 μl of gene expression assay and 2 μl of cDNA. The gene expression assays used were: *hsMC1R (*NM_002386.3): Hs00267168-S1; *hsMC2R (*NM_000529.2): Hs003000820-S1; *hsMC3R (*NM_019888.3): Hs01562847-S1; *hsMC4R (*NM_005912.2): Hs00271877_S1; *hsMC5R (*NM_005913.1): Hs00271882_S1; *hsGAPDH* (NM_002046.4): Hs02758991_g1.

### Binding studies

The human MC1R, MC3R, MC4R and MC5R were cloned by PCR and subcloned into the pcDNA3 expression vector, as previously described by Conde-Frieboes et al. [43]. Cells stably expressing the human MCRs were generated by transfecting the expression vector into BHK570 cells (ATTC) and using 1 mg/mL G418 to select for stable clones. The stable cell lines were cultured in DMEM with glutamax, 10 % FCS, 1 % pen/strep, and 1 mg/mL G418 at 37 °C and 5 % CO_2_. Binding affinities were obtained in vitro using ^125^I-NDP-α-MSH binding to membranes from recombinant BHK570 cells expressing the relevant human melanocortin receptor. Test compounds were dissolved to a 4 mM concentration in DMSO and diluted in a receptor specific binding buffer. A fixed concentration of ^125^I-NDP-α-MSH and varying concentrations (10 μM–1 pM) of non-labelled competing test compound were added to the cells. A filtration system was employed to separate bound from unbound radioligand.

### NEFA and glycerol release in differentiated human adipocytes and WAT explants

Induction of NEFA and glycerol release in adipocytes and intact adipose tissue explants was used as a measure of lipolysis with β-adrenergic agonist isoproterenol as a positive control as previously described [35]. When using propranolol, 10 μM was used as final concentration. 250 mg of human adipose tissue was placed in 15 mL tubes, immediately covered with 2.5 mL of preheated (37 °C) hyclone media and preincubated for 24 h. After 24 h, the media was replenished and explants were stimulated with the indicated melanocortin peptide for 3 h before harvesting the supernatant for NEFA and glycerol analysis. In house testing has shown no indications of hypoxia when stimulating explants (data not shown). All test compounds were dissolved to 4 mM in DMSO (highest DMSO concentration of 0.25 % in the assay) and diluted in DPBS buffer + 2 % bovine serum albumin (BSA). Human subcutaneous adipocytes purchased from Zenbio Inc., were used in stimulation of lipolysis (n = 4). These adipocytes have been isolated from obese males. When receiving the cells (and partially during transportation), the cells was differentiated from a pre-state to a mature state before the cells are used for experiments. The cells were handled according to the description from ZenBio Inc. Two hours was chosen as the optimal incubation time span for primary adipocytes, based on similar experiments in other species [35]. The release of NEFA proved to be a reproducible measure of lipolysis, though potential re-esterification of NEFAs would underestimate the degree of MCR mediated lipolysis. Indeed, a potent release of NEFA was stimulated by positive control isoproterenol in both adipocytes and intact adipose tissue explants. Measures of melanocortin-stimulated glycerol release were added in explant lipolysis studies.

### Data analysis and statistics

Data from the NEFA and the glycerol assay were obtained using the protocol supplied by the manufactures. Released NEFAs were analyzed in Graphpad Prism and one-tailed *t* test was used to calculate statistical significance between 2 groups (*p < 0.05, **p < 0.01, ***p < 0.001, no * indicates insignificance). K_i_ values in bindings studies were calculated as K_i_ = IC50/1 + [radioligand]/K_d_. Data from binding studies was analyzed in Graphpad Prism.

## Results

### Binding data of known melanocortin analogues to MC1R, MC3R, MC4R and MC5R

In order to assess MCR agonist specificity, competition binding curves of α-MSH, ACTH, PG-901 and LY2112688 were obtained using cells overexpressing the human MCRs (Table 1). MC2R is acknowledged to be activated only by ACTH [23], and for this reason, binding studies were not performed on this receptor. Our binding data support that remaining MCRs bind ACTH with relatively high affinities (MC1R > MC4R > MC3R > MC5R), which is also consistent with previous studies [44]. Results obtained support present literature on human MCRs, stating that α-MSH is a non-selective agonist that binds to all MCRs [45]. LY2112688 has previously been studied by Greenfield et al., who found that the agonist was highly selective for MC4R, having an increased affinity for MC4R over MC1R of a factor more than 30 and over MC3R of a factor of more than 100 [46]. We support that LY2112688 is selective for human MC4R (K_i_ = 0.13 ± 0.01), but we find the binding rank order to be MC4R > MC3R > MC1R. Notably, the affinity of LY2112688 for MC1R is 165 ± 51 nM, which is in the range of concentrations used in lipolysis assay. In the literature, PG-901 binds MC5R (agonist), as well as MC3R and MC4R (antagonist) with high affinity [47], which is consistent with our studies.

**Table 1:** Binding (K_i_) of melanocortin peptides to human MCRs: Indicated values are an average from repeated independent experiments (±SEM)

Test compound	hMC1R	hMC3R	hMC4R	hMC5R
	*K*_*i*_ (nM)	*K*_*i*_ (nM)	*K*_*i*_ (nM)	*K*_*i*_ (nM)
LY2112688	165 ± 51 (3)	74 ± 27 (3)	0.13 ± 0.01(3)	1713 ± 686 (3)
PG-901	2700 ± 153 (3)	11 ± 0.5 (4)	0.08 ± 0.01 (3)	1.7 ± 0.6 (4)
α-MSH	1.5 ± 0.4 (18)	46 ± 5 (9)	10.16 ± 3.4 (3)	150 ± 30 (7)
ACTH	0.09 ± 0.01 (3)	11.1 ± 1.5 (5)	1.2 ± 0.2 (3)	46 ± 9.5 (5)

K_i_ values were calculated from competition binding curves of α-MSH, PG-901 and LY2112688 obtained on cells expressing MC1, 3, 4 and 5R as described using a fixed concentration of I^125^-NDP-α-MSH and increasing amounts of non-labelled competing peptides (1 pM–10 μM)

### NEFA release in subcutaneous explants from human white adipose tissue

**Fig. 1:**
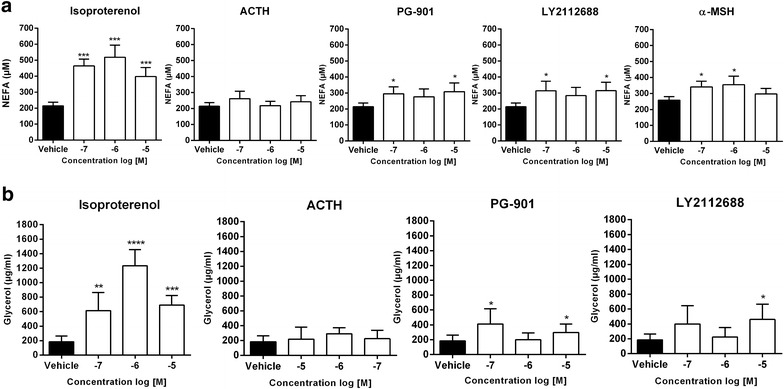
Non-esterified fatty acid (NEFA) and glycerol release in intact adipose tissue explants from human. **a** NEFA release was measured after stimulation with increasing doses of isoproterenol or the indicated melanocortin peptide (100 nM–10 μM) in subcutaneous explants from non-obese females. The mean of 6 independent experiments (α-MSH, 3 independent experiments) in triplicate wells (mean ± SEM) are shown. **b** Glycerol release was measured after stimulation with increasing doses of isoproterenol or the indicated melanocortin peptide (100 nM–10 μM) in subcutaneous explants from non-obese females. The mean of 2 independent experiments in triplicate wells (mean ± SEM) are shown. Unpaired one-tailed t test was used to calculate statistical significance (*p < 0.05, **p < 0.01, ***p < 0.001 vs. vehicle)

To examine the function of MCRs in human adipose tissue, we investigated the ability of MCR agonists to stimulate lipolysis in intact explants (Fig. 1). Isoproterenol, which is known to stimulate release of NEFA in human WAT through activation of the β-adrenergic receptors, was used as a positive control. Indeed, the release of NEFA was increased by 114 % using 100 nM isoproterenol compared to vehicle (Fig. 1a). Furthermore, 100 nM α-MSH, MC4R-selective agonist LY2112688 and MC5R-selective agonist PG-901 stimulated a low, though significant, release of NEFA (32, 46 and 38 % increase compared to the vehicle). PG-901 and LY2112688 also stimulated release of glycerol. Compared to isoproterenol, α-MSH, LY2112688 and PG-901 stimulated lipolysis with a relatively lower E_max_. Notably, ACTH failed to stimulate NEFA and glycerol release in human WAT explants (Fig. 1a, b). Binding results obtained in this study support that LY2112688 has high affinity (K_i_ = 0.13 ± 0.01 nM) for the human MC4R and lower affinity for other human melanocortin receptors. However, as LY2112688 bind to MC1R with a K_i_ = 165 ± 51 nM, the LY2112688-stimulation of lipolysis may partially be mediated by MC1R considering the concentration used.

### MCRs have no lipolytic effect in differentiated adipocytes from obese human subjects

**Fig. 2:**
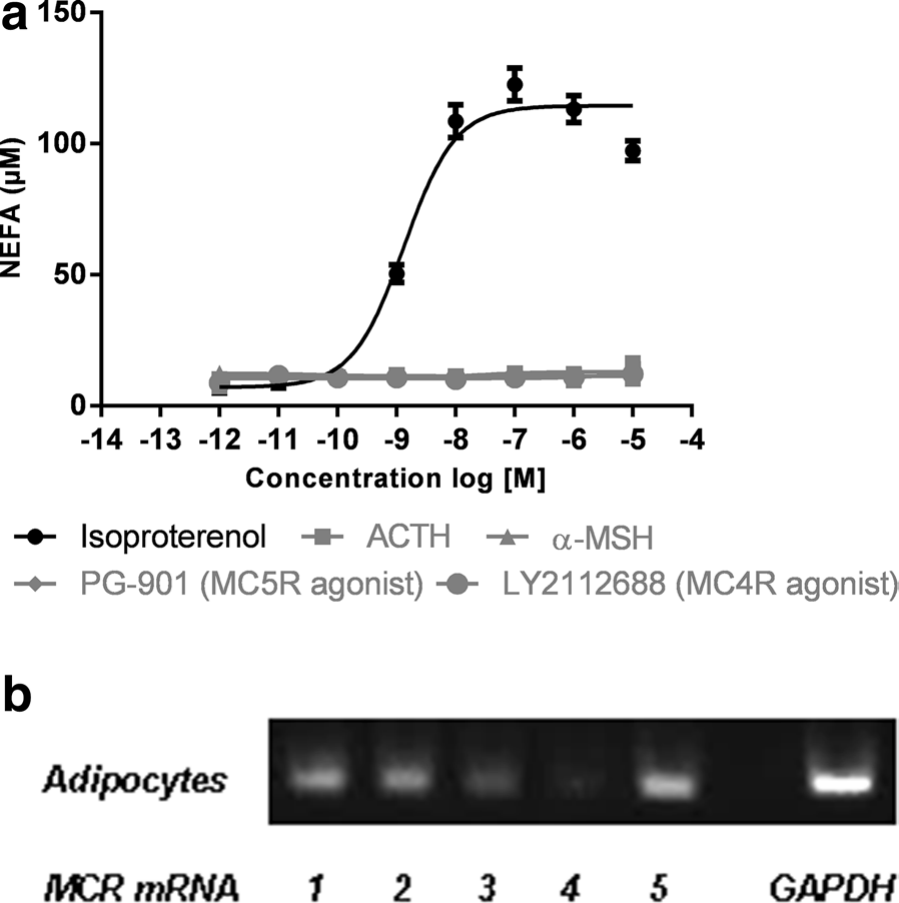
Non-esterified fatty acid (NEFA) release in human subcutaneous adipocytes: **a** Adipocytes purchased from ZenBio were incubated with increasing concentrations (1 pM–10 μM) of positive control isoproterenol (EC_50_ = 1.46 ± 0.34 nM), ACTH, α-MSH, PG-901 and LY2112688 (n = 4). NEFA release was measured as described. Experiments were carried out in duplicates. Data are shown as average values ± SEM. **b** MC1-5R and GAPDH mRNA expression was measured in same adipocytes (n = 3 pooled). cDNA was synthesized and mRNA levels determined by standard PCR (cDNA 1:10 from 1 μg RNA, 45 cycles)

We investigated the ability of the MCR agonists to stimulate lipolysis in subcutaneous adipocytes from obese males (purchased from Zenbio). While isoproterenol induced a potent release of NEFA in human subcutaneous adipocytes (EC_50_ = 1.64 ± 0.34 nM) α-MSH, ACTH, PG-901 and LY2112688 all failed to stimulate NEFA release (Fig. 2). We also stimulated visceral adipocytes with the same results as in Fig. 2 (data not shown). Standard PCR showed that the expression of MC3R and MC4R mRNA is low in subcutaneous adipocytes in the conditions used, whereas the expression of MC1R, MC2R and MC5R is higher (Fig. 2).

### The partial lipolytic effect of MC5R in explants is dependent on postsynaptic innervation

**Fig. 3:**
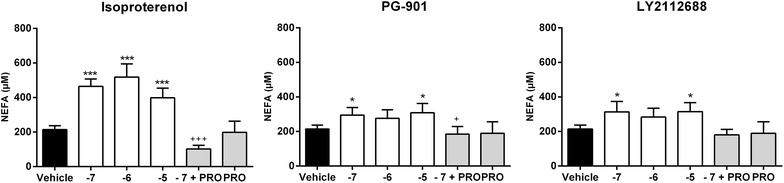
Non-esterified fatty acid (NEFA) release in human white adipose tissue (WAT) explants ± propranolol: NEFA was measured after stimulation with indicated melanocortin peptide analogue (100 nM–10 μM) in subcutaneous explants from female human ± 10 μM β-adrenergic antagonist propranolol (PRO). The mean ± SEM of 3 independent experiments in triplicate wells are shown. Unpaired one-tailed t test was used to calculate statistical significance (*p < 0.05, **p < 0.01, ***p < 0.001 vs. vehicle; ^+^p < 0.05, ^++^p < 0.01, ^+++^p < 0.001 vs. −7)

Since we only observed a lipolytic effect in intact adipose tissue and not in adipocytes, we examined whether MCR-mediated lipolysis involves neuronal interaction. Hence, we co-incubated PG-901 and LY2112688 with β-adrenergic antagonist propranolol in intact adipose tissue (Fig. 3). Use of propranolol significantly decreased the release of NEFA stimulated by isoproterenol and MC5R selective agonist PG-901. This result suggests that the lipolytic effect of MC5R may be mediated by noradrenalin released from postsynaptic nerve fibers innervating the adipose tissue. Use of propranolol did not significantly decrease the release of NEFA stimulated by MC4R-selective agonist LY2112688, although there is a clear trend.

## Discussion

Melanocortin receptors are important central regulators of energy metabolism and feeding behavior. These receptors also have a direct lipolytic effect in rodent [35, 37, 38] and rabbit [39] adipose tissues. The storage and release of lipids in adipose tissue is a highly regulated process, which play an essential role in whole body homeostasis. A systemic abundance of both endogenous melanocortin agonists and antagonists have been established [1, 18, 48] and MCRs are expressed in peripheral tissues relevant for lipid metabolism. Hoch et al. established MC1R mRNA expression in adipose tissue [26], Chhajlani et al. identified MC1R and MC5R in omental adipose tissue [41] and Chagnon et al. linked MC4R and MC5R with an obese-related phenotype [40]. The presence of a peripheral melanocortin system in adipose tissue is supported by results from this study.

In this study, we show that α-MSH, MC5R selective agonist PG-901 and MC4R selective agonist LY2112688 significantly stimulate release of NEFA in intact adipose tissue. Notably, as LY2112688 bind to MC1R with a K_i_ = 165 ± 51 nM, the LY2112688-stimulation of lipolysis may partially be mediated by MC1R considering the concentration used. A recent study by Rodrigeus et al. showed that α-MSH-stimulated MC5R regulates both lipolysis and re-esterification in murine 3T3-L1 cells, though the global effect is a decrease in adipocyte fat mass [49]. This may be relevant in terms of the MC5R-mediated lipolysis seen is this study, as some of the released free fatty acids (FFAs) may be re-esterified and taken up by the adipocyte again. However, as PG-901 and LY2112688 also stimulated release of glycerol there seems to be a persistent lipolytic effect of these compounds.

**Fig. 4:**
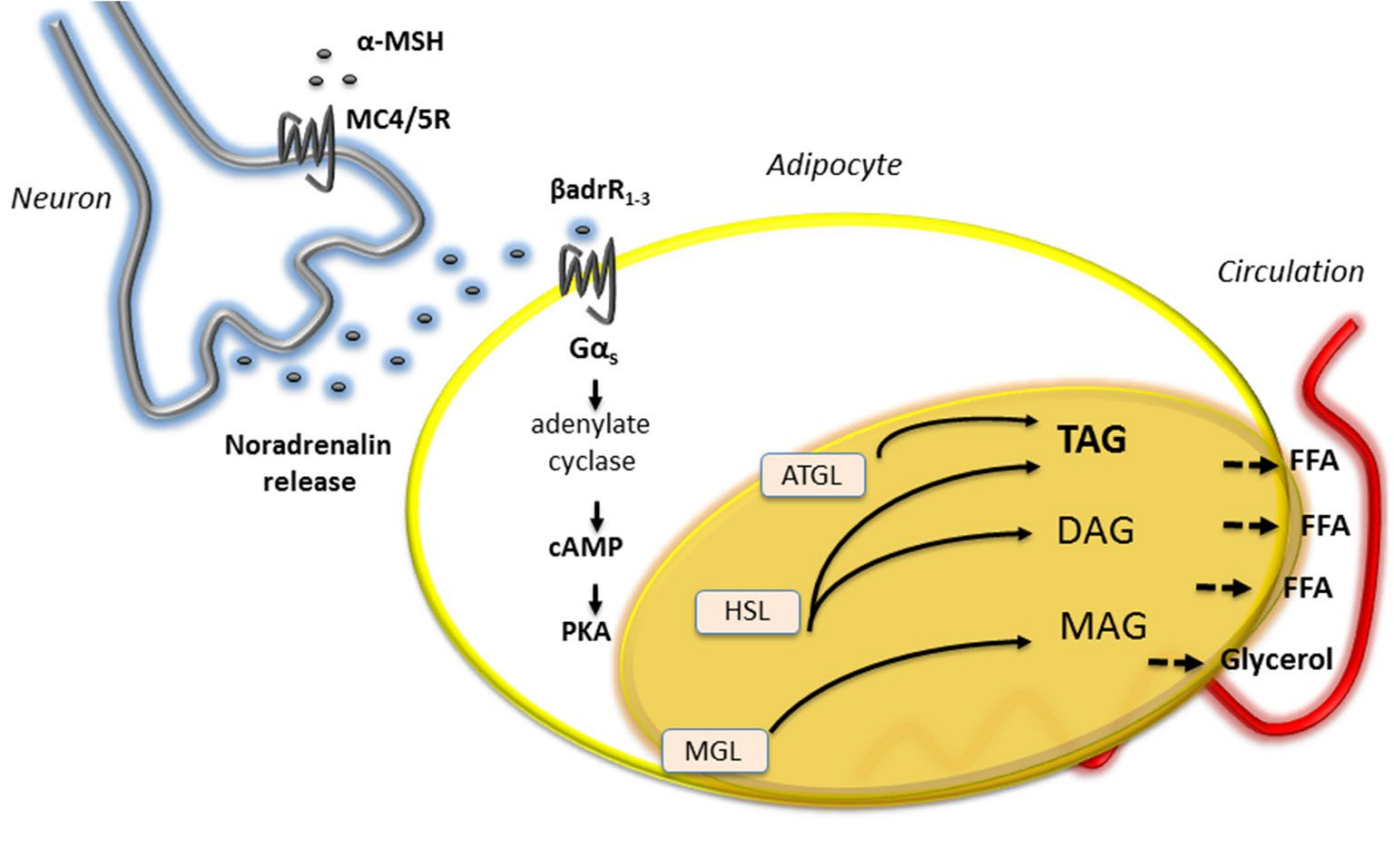
An overview of melanocortin receptor (MCR)-mediated lipolysis in intact human white adipose tissue (WAT). It is hypothesised that MC4/5R mRNA is expressed on nerve terminals innervating WAT. α-MSH from the circulation or from ectopic production binds to MC4/5R, which increase the release of noradrenalin from the terminal. Stimulated β_1–3_-adrenergic receptors couple to Gαs and downstream adenylate cyclase leading to increased intracellular cAMP and activation of protein kinase A (PKA). The induction of lipolysis mediated by MC4R and MC5R is suggested to be dependent on neuronal innervation via noradrenalin release, since co-incubation of propranolol (non-selective β-adrenergic receptor antagonist) inhibits NEFA release

The lipolytic response of PG-901 was significantly decreased when using β-adrenergic antagonist propranolol, suggesting that the effect may be dependent on neuronal innervation via noradrenalin release (Fig. 4). Propranolol is a non-selective β-adrenergic antagonist that is used as a golden standard for inhibition of neuronal innervation. However, it should be taken into account, that there may be downstream MCR mechanisms of propranolol that are unknown, which is a precaution using this antagonist. Depleting the explants with reserpine before stimulation could add additional confidence to the result of propranolol. Centrally expressed MC4R in the hypothalamus and the brainstem has in several studies been shown to regulate peripheral metabolism through sympathetic nerve innervation. Through innervation, CNS-MC4R increases lipolysis in WAT [11, 12], insulin sensitivity in muscle [12, 13], thermogenesis in brown adipose tissue (BAT) [10], fatty acid oxidation in liver [14] and decreases insulin release in pancreatic β-cells [15] as mentioned previously. Likewise, Wellhöner et al. showed that intranasal MSH/ACTH(4-10) administration increased glycerol concentrations in WAT, presumably mediated by centrally expressed MC4R [50]. However, the mode of action suggested in the present study is not a CNS-MCR regulated pathway. Instead, we suggest that peripherally expressed MC5R co-localized with post-synaptic neurons directly in adipose tissue regulate low grade lipolysis. Notably, the central and peripheral regulation of MCR-mediated lipolysis may not exclude each other. α-MSH has been shown to inhibit proliferation and adipogenesis in human preadipocytes mediated by MC1R, which is antagonized by agouti [24]. Considering the above, a lipolytic capacity of α-MSH in human adipose tissue seems plausible. If stimulation of MC5R/MC4R in adipose tissue leads to increased levels of plasma FFA and glycerol, these lipids may likely be absorbed and metabolized in the liver. This may lead to increased hepatic lipid deposition and potentially hepatic insulin resistance. However, if energy expenditure simultaneously is increased by melanocortins these lipids may also serve as energy substrates to be utilized in muscle. Notably, An et al. has shown that MC5R mediates α-MSH-stimulated fatty acid oxidation in muscle tissue [33]. Additional studies are needed to determine where in fact the released lipids are absorbed and metabolized.

We find no lipolytic capacity of ACTH in either adipocytes or intact human adipose tissue, which is supported by other studies [51, 52]. ACTH is a larger peptide than both α-MSH and the synthetic melanocortin compounds used in this study. It is possible that the lack of lipolytic response ex vivo observed when stimulating with ACTH is due to the size of the peptide. Opposing results have been reported by Xue et al. who stimulated lipolysis using ACTH in adipocytes isolated from subcutaneous adipose tissue of non-diabetic human [53].

We, and others, have previously shown that the melanocortin system is involved in peripheral regulation of adipocyte metabolism in mice and has direct lipolytic capacity in addition to central regulation. Indeed, ACTH and α-MSH were reported to have a lipolytic capacity in primary mouse adipocytes, which is mediated by MC2R and MC5R [35, 37, 38]. This result was not found in human adipocytes, which suggests essential species differences in the melanocortin system between mice and humans. The lack of lipolytic response seen in adipocytes is supported by previous studies by Hoch et al. [52]. Hoch et al. induced a weak cAMP response using NDP-α-MSH in mesenchymal stem cell-derived adipocytes. However, they did not find an effect on lipolysis in either these cells or in intact adipose tissue explants [52].

Further studies are needed in order to establish the relevance of the melanocortin receptor accessory proteins, MRAP and MRAP2, in intact adipose tissue versus adipocytes. Studies have shown that all MCRs may employ an accessory protein, which modulates the functionality of the receptor [54]. It may be that MRAP/MRAP2 mRNA levels are not equally expressed in the stroma vascular fraction (SVF) and in isolated adipocytes.

Collectively, the E_max_ induced by melanocortin peptides in intact adipose tissue explants is small compared to isoproterenol. It is possible that the melanocortin system has other functions in adipose tissue than induction of lipolysis. The SVF of adipose tissue consists of cells such as preadipocytes, mesenchymal stem cells, several types of inflammatory cells, as well as nerve fibers and blood vessels. α-MSH inhibits TNF-α-induced NF-κB in immune cells [55], as well as in a subset of other human cells such as melanocytes, fibroblasts, keratinocytes, endothelial cells and Schwann cells of the peripheral nervous system [56]. Suppression of NF-κB potentially controls the expression of 150 genes including cytokines for which reason the involvement of the melanocortins in inflammation is potentially profound [56]. It has been shown that the inflammatory effects of the melanocortin system are regulated through mainly MC1R and MC3R [26, 56]. Hence the primary function of MCRs in human adipose tissue may be inflammatory rather than lipolytic [52].

We find that various selective and non-selective melanocortin peptides significantly induce low-grade lipolysis in intact human adipose tissue. The physiological relevance of these receptors in terms of human adipose tissue lipolysis is probably minimal. The plasma level of circulating endogenous α-MSH and ACTH is on average reported to be 11 pmol/l (~0.011 nM) and 14.5 pmol/l (~0.015 nM) [27]. These concentrations are not compatible with the concentration of endogenous and synthetic melanocortin receptor ligands used in this study. However, even the physiological concentration of endogenous α-MSH circulating in the human body is below the EC_50_ values for the peripheral MCRs [57]. Does circulating α-MSH have any physiological relevance for adipose tissue metabolism? In terms of circulating endogenous melanocortin peptides the answer is probably no. However, in terms of developing an anti-obesity therapeutic drug with selective MC4R/MC5R properties directed against the effects on satiety and energy expenditure, the answer may be yes. In addition, paracrine POMC-secretion/ectopic POMC-production could increase the concentration and exposure of ACTH and α-MSH to MCRs in peripheral tissues such as white adipose tissue.

## Conclusion

Selective and non-selective melanocortin peptides significantly induce low-grade lipolysis in intact human adipose tissue but not in adipocytes. The lipolytic response of MC5R was decreased in intact human white adipose tissue when co-treating with β-adrenergic antagonist propranolol, suggesting that the effect may be dependent on neuronal innervation via noradrenalin release. These effects may be physiologically relevant, when developing an anti-obesity therapeutic drug with selective MC4R/MC5R properties.

## Abbreviations

AgRP: Agouti-related peptide
ASIP: Agouti signaling protein
BMI: Body mass index
FFA: Free fatty acids
MCR: Melanocortin receptor
MRAP: Melanocortin receptor-associated protein
NEFA: Non-esterified fatty acid
POMC: Proopiomelanocortin
PRO: Propranolol
SVF: Stroma vascular fraction
WAT: White adipose tissue
